# Supplementary material for: Proteomics Approach of Rapamycin Anti-Tumoral Effect on Primary and Metastatic Canine Mammary Tumor Cells In Vitro
Source: Molecules. 2021 Feb 25;26(5):1213. doi: 10.3390/molecules26051213 (PMC7956669; doi:10.3390/molecules26051213)
Supplement: Supplementary file 1 [file molecules-26-01213-s001.pdf]

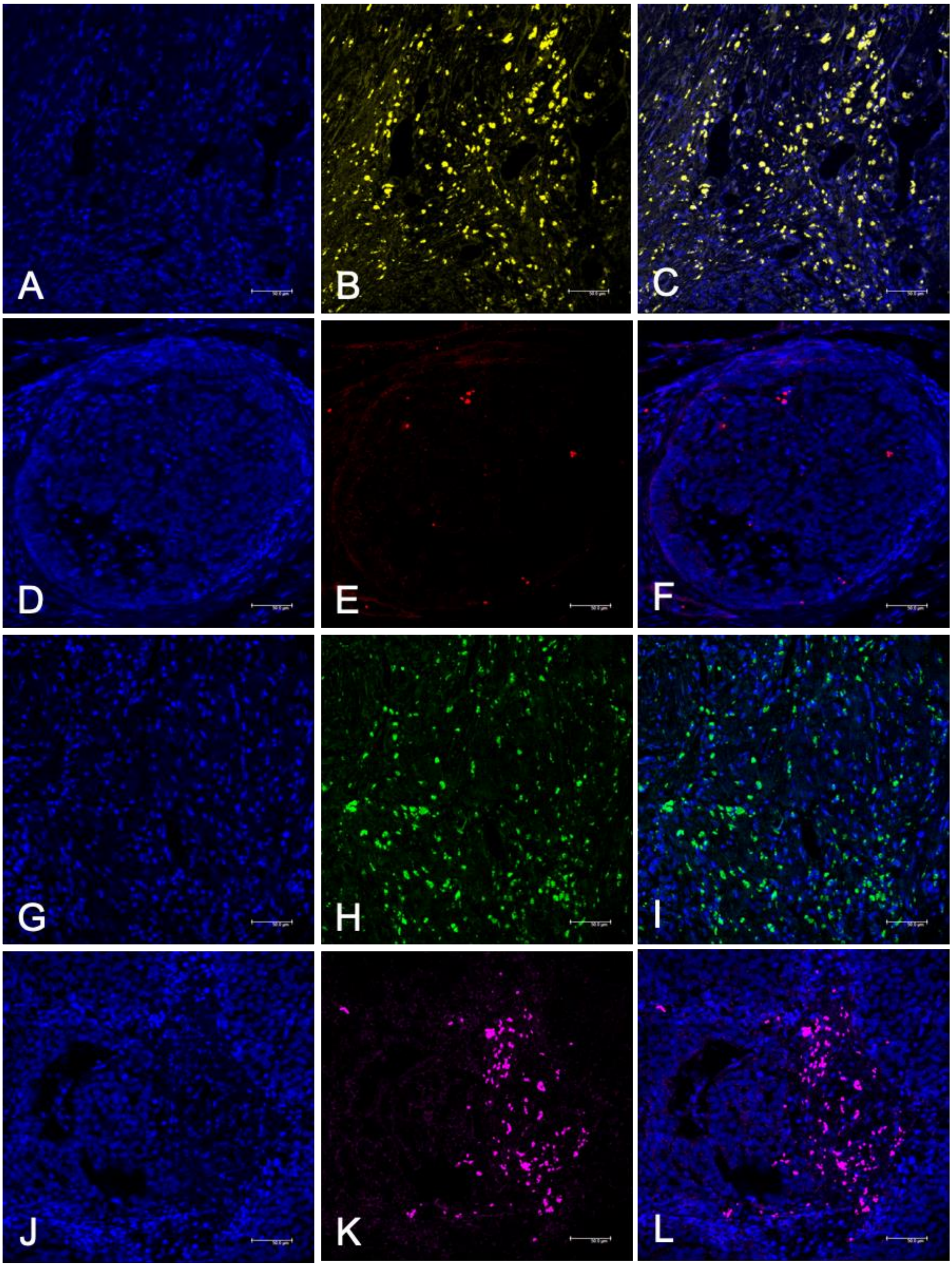

Supplementary Figure S1. Immunofluorescence of canine primary breast tumor. Staining with DAP-I (A, D, G and J), staining with FITC (B, E, H and K) and Merge (C, F, I and L). Positive expression for PTEN (C), mTOR (F), AKT (I) and 4EBP1 (L) proteins.

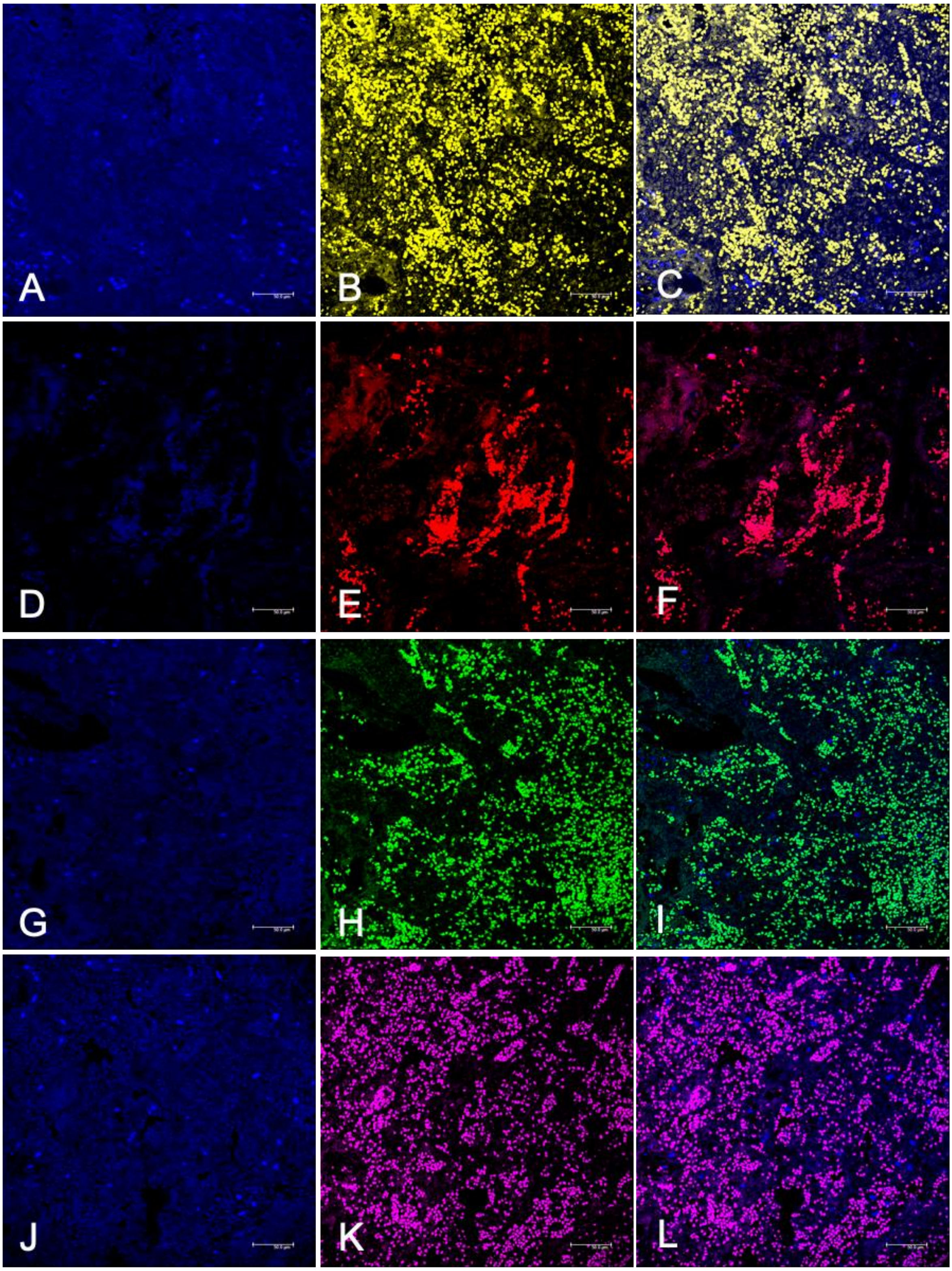

Supplementary Figure S2. Immunofluorescence of canine metastasis tumor. Staining with DAP-I (A, D, G and J), staining with FITC (B, E, H and K) and Merge (C, F, I and L). Positive expression for PTEN (C), mTOR (F), AKT (I) and 4EBP1 (L) proteins.

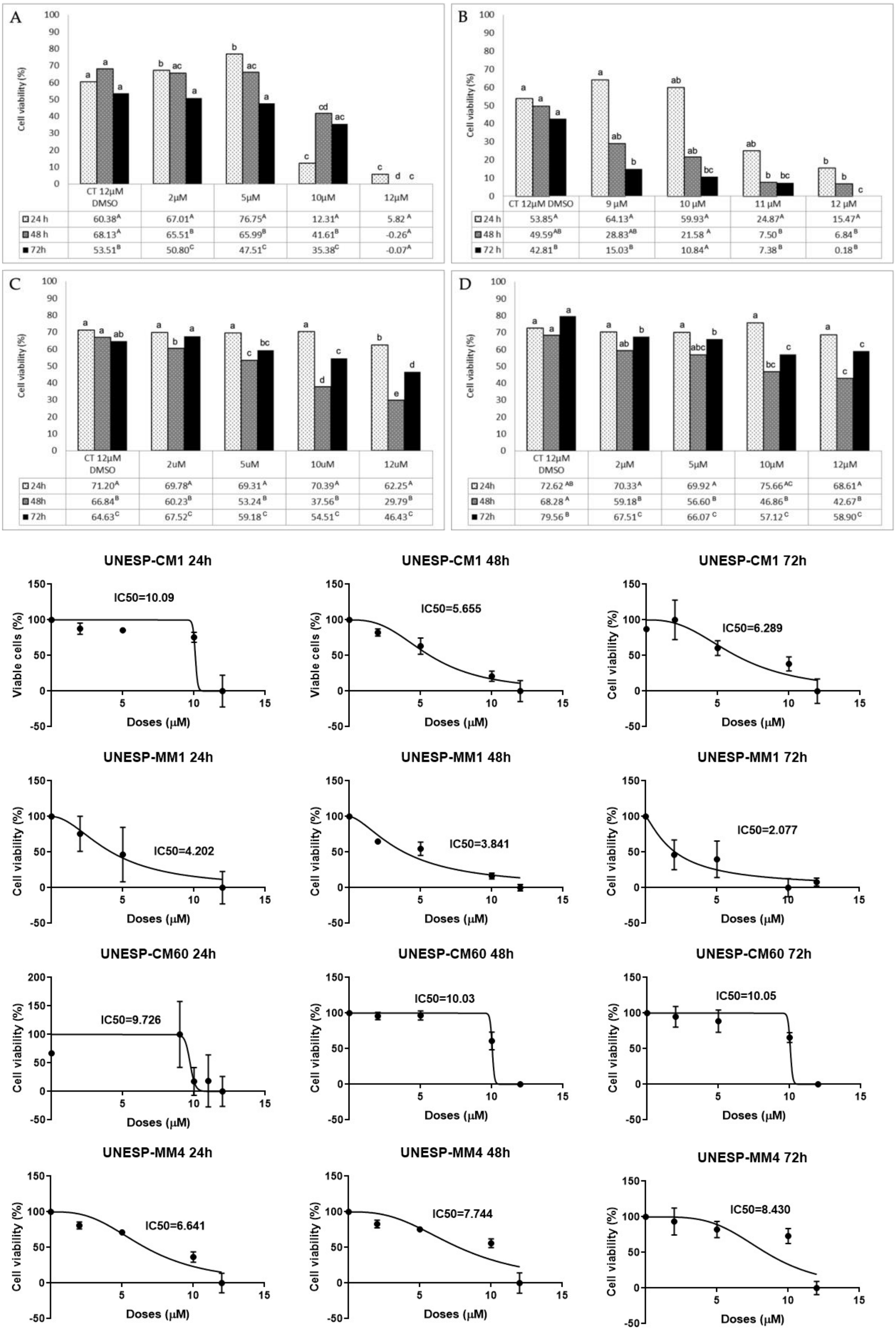

Supplementary Figure S3. Cell viability percentage of primary tumors and their respective metastasis treated with different doses of rapamycin in MTT assay and their IC$_{50}$ value, evaluated in 24, 48 and 72 h. A: UNESP-CM60. B: UNESP-MM4. C: UNESP-CM1. D: UNESP-MM1. Different lowercase letters in bars indicate statistical difference between the rapamycin doses at the same

time. Different capital letters in columns of data label indicate the statistical difference between times in the same dose treatment.

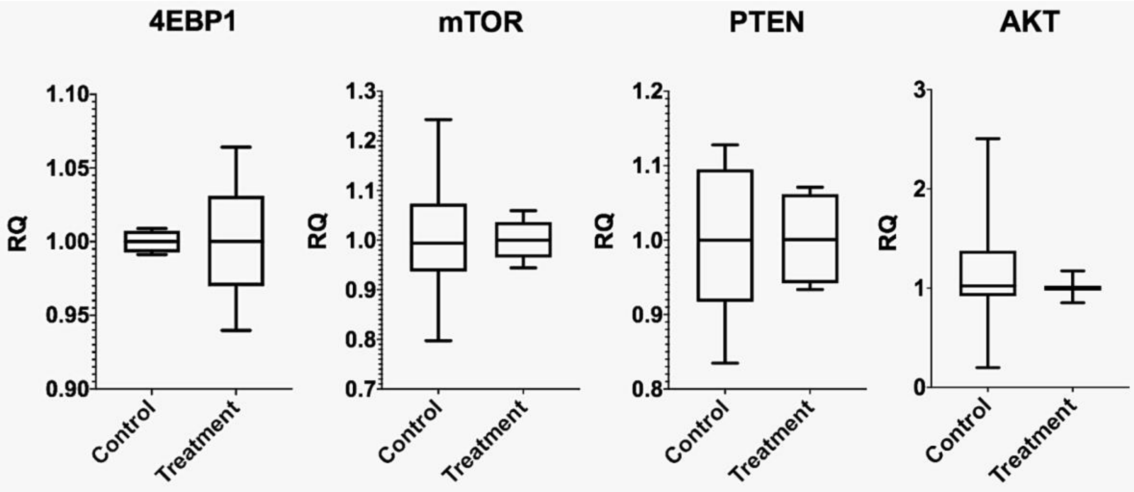

Supplementary Figure S4. Gene expression of *AKT*, *PTEN*, *mTOR* and *4EBP1* in control group *versus* rapamycin treatment group.

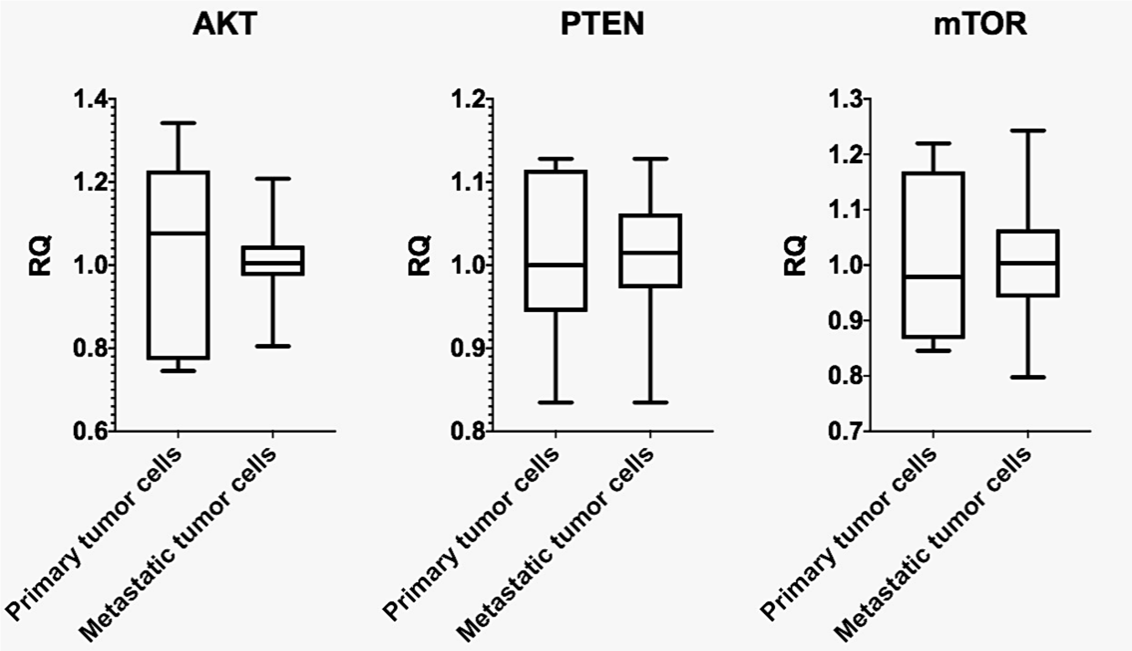

Supplementary Figure S5. Gene expression of *AKT*, *PTEN* and *mTOR* of primary tumor cells group *versus* metastatic cells group.

Supplementary Table S1. Mean ± standard error of protein expression for PTEN, mTOR, AKT and 4EBP1 in primary mammary gland tumors and their respective metastases.

Antibody	Primary tumors (n = 2)	Metastases (n = 2)
PTEN (%)	2.36 ± 0.23	24.67 ± 4.38
mTOR (%)	0.12 ± 0.03	4.05 ± 1.67
AKT (%)	2.32 ± 0.73	9.22 ± 4.52
4EBP1 (%)	1.25 ± 0.01	17.55 ± 2.31

Supplementary Table S2. Median and range values from RT-qPCR gene expression for control *versus* rapamycin treatment groups.

Gene	Median (Control)	Median (Rapamycin Treatment)	Range (Control)	Range (Rapamycin Treatment)
4EBP1	1	1	0.017	0.125
AKT	1.023	1	2.305	0.321
mTOR	0.9936	1	0.445	0.1116
PTEN	1	1.001	0.293	0.138

Supplementary Table S3. Median and range values from RT-qPCR gene expression for primary tumor cells *versus* metastatic tumor cells groups.

Gene	Median (Primary tumor cells)	Median (Metastatic cells)	Range (Primary tumor cells)	Range (Metastatic cells)
AKT	1.076	1.005	0.596	0.233
mTOR	0.9787	1.004	0.374	0.445
PTEN	1	1.015	0.293	0.293

Supplementary Table S4. Proteins identification and gene ontology of control and rapamycin group.

Name	ID*	Gene	Gene Ontology		
			**Molecular function**	**Biological process**	**Cellular component**
Phosphoglycerate mutase	E2RT65	PGAM1	- Bisphosphoglycerate mutase activity<br>-Phosphoglycerate mutase activity<br>- Protein kinase binding	- Glycolytic process<br>- Regulation of glycolytic process<br>- Regulation of pentose-phosphate shunt<br>- Respiratory burst	- Cytosol
Myotrophin	Q863Z4	MTPN	- Promotes dimerization of NF-kappa-B subunits and regulates NF-kappa-B transcription factor activity<br>- Promotes growth of cardiomyocytes, but not cardiomyocyte proliferation<br>- Promotes cardiac muscle hypertrophy<br>- Plays a role in the regulation of the growth of actin filaments<br>- Inhibits the activity of the F-actin-capping protein complex formed by the CAPZA1 and CAPZB heterodimer	- Positive regulation of cardiac muscle hypertrophy<br>- Positive regulation of cell growth<br>- Positive regulation of macromolecule biosynthetic process<br>- Positive regulation of NF-kappaB transcription factor activity<br>- Positive regulation of protein metabolic process<br>- Regulation of barbed-end actin filament capping<br>- Regulation of cell size	- Cytoskeleton<br>- Cytosol<br>- Nucleus<br>- Axon<br>- Cytoplasm<br>- Perinuclear region of cytoplasm
L-lactate dehydrogenase	E2R761	LDHB	- L-lactate dehydrogenase activity	- Carbohydrate metabolic process<br>- Carboxylic acid metabolic process	- Cytoplasm
Nucleolin	F1Q0B0	NCL	- RNA binding	- Positive regulation of mRNA splicing, via spliceosome<br>- Regulation of RNA metabolic process	- Nucleus<br>- Spliceosomal complex<br>- Ribonucleoprotein complex
14_3_3 domain-containing protein	F1PBL1	YWHAZ	--	--	--
Annexin A2	Q6TEQ7	ANXA2	- Calcium channel activity<br>- Calcium-dependent phospholipid binding<br>- Calcium ion binding<br>- Cytoskeletal protein binding<br>- Phosphatidylinositol-4,5-bisphosphate binding<br>- Phosphatidylserine binding<br>- Phospholipase A2 inhibitor activity<br>- Protease binding<br>- Rab GTPase binding<br>- Virion binding	--	- Endosome<br>- Basement membrane<br>- Extracellular space Nucleus<br>- Plasma Membrane<br>- Cytoplasm<br>- Exocytic vesicle<br>- Vesicle
Succinate dehydrogenase (quinone)	Q0QF09	SDHA	- Flavin adenine dinucleotide binding<br>- Succinate dehydrogenase (ubiquinone) activity	- Electron transport chain<br>- Tricarboxylic acid cycle	- Membrane
Carboxypeptidase	F1PSP6	SCPEP1	- Serine-type carboxypeptidase activity	- Negative regulation of blood pressure<br>- Positive regulation of blood vessel diameter<br>- Proteolysis involved in cellular protein catabolic process	--
Cytochrome c	P00011	CYCS	- Electron transporter, transferring electrons from CoQH2-cytochrome c reductase complex and cytochrome c oxidase complex activity	- Apoptotic process<br>- Mitochondrial electron transport, cytochrome c to oxygen<br>- Mitochondrial electron transport, ubiquinol to cytochrome c	- Cytosol<br>- Mitochondrial intermembrane space<br>- Nucleus

			- Heme binding<br>- Metal ion binding	- Positive regulation of cysteine-type endopeptidase activity involved in apoptotic process	- Respirasome
GST class-pi	F1Q0J0	GSTP1	- Glutathione transferase activity	- Glutathione metabolic process	- Cytosol
Malate dehydrogenase	F1PYG8	MDH2	- L-malate dehydrogenase activity	- Carbohydrate metabolic process<br>- Malate metabolic process<br>- Tricarboxylic acid cycle	- Mitochondrion<br>- Cytoplasm
Nucleoside diphosphate kinase A	Q50KA9	NME1	- ATP binding<br>- Metal ion binding<br>- Nucleoside diphosphate kinase activity	- Cell differentiation<br>- CTP biosynthetic process<br>- Endocytosis<br>- GTP biosynthetic process<br>- Nervous system development<br>- Purine nucleotide metabolic process<br>- Pyrimidine nucleotide metabolic process<br>- Regulation of apoptotic process<br>- UTP biosynthetic process	- Nucleus<br>- Citoplasm
Glucosidase II alpha subunit	F1Q4J0	GANAB	- Carbohydrate binding<br>- Hydrolase activity, hydrolyzing O-glycosyl compounds	- Carbohydrate metabolic process	--
Rab GDP dissociation inhibitor	F1P8L7	GDI2	- GTPase activator activity<br>- Rab GDP-dissociation inhibitor activity<br>- Rab GTPase binding	- Protein transport<br>- Small GTPase mediated signal transduction<br>- Vesicle-mediated transport	- Cytoplasm
MAPK activated protein kinase 5	F1PBJ8	MAPKAPK 5	- Aldehyde dehydrogenase (NAD+) activity<br>- ATP binding<br>- Protein kinase activity	--	--
E2RJ06	40S ribosomal protein SA	E2RJ06 (obsolete) PI0002749154	RPSA	--	--
Obg-like ATPase 1	F1PI87	OLA1	- ATPase activity<br>- ATP binding<br>- GTP binding<br>- Ribosomal large subunit binding<br>- Ribosome binding	--	- Nucleolus<br>- Cytoplasm
Dihydropyrimidinase like 2	F1P9U4	DPYSL2	- Hydrolase activity, acting on carbon-nitrogen (but not peptide) bonds, in cyclic amides<br>- Microtubule binding<br>- Protein kinase binding	- Axon guidance<br>- Brain development<br>- Cytoskeleton organization<br>- Regulation of axon extension	- Cytosol<br>- Plasma Membrane<br>- Axon<br>- Dendrite<br>- Neuronal cell body
Phosphoglucomutase 1	F1PUL4	PGM1	- Magnesium ion binding<br>- Phosphoglucomutase activity	- Galactose catabolic process<br>- Glucose metabolic process<br>- Glycogen biosynthetic process	- Cytosol
Calreticulin	F6UYJ9	CALR	- Androgen receptor binding<br>- Calcium ion binding<br>- Carbohydrate binding<br>- Integrin binding	- Cellular senescence<br>- Cortical actin cytoskeleton organization<br>- Endoplasmic reticulum unfolded protein response<br>- Negative regulation of cell cycle arrest	- Endoplasmic reticulum lumen

			- mRNA binding<br>- Ubiquitin protein ligase binding<br>- Unfolded protein binding	- Negative regulation of intracellular steroid hormone receptor signaling pathway<br>- Negative regulation of neuron differentiation<br>- Negative regulation of retinoic acid receptor signaling pathway<br>- Negative regulation of transcription by RNA polymerase II<br>- Negative regulation of translation<br>- Negative regulation of trophoblast cell migration<br>- Peptide antigen assembly with MHC class I protein complex<br>- Positive regulation of cell population proliferation<br>- Positive regulation of dendritic cell chemotaxis<br>- Positive regulation of endothelial cell migration<br>- Positive regulation of gene expression<br>- Positive regulation of NIK/NF-kappab signaling<br>- Positive regulation of phagocytosis<br>- Positive regulation of substrate adhesion-dependent cell spreading<br>- Protein export from nucleus<br>- Protein folding<br>- Protein localization to nucleus<br>- Protein stabilization<br>- Regulation of meiotic nuclear division	
Biliverdin reductase B	E2QVU9	BLVRB	--	--	--
AHNAK nucleoprotein	J9P969	AHNAK	- Regulation of RNA splicing	--	- Plasma Membrane<br>- Costamere<br>- Cytoplasm
Pyruvate dehydrogenase E1 component subunit beta	E2R268	PDHB	- Pyruvate dehydrogenase (acetyl-transferring) activity	- Acetyl-CoA biosynthetic process from pyruvate	- Mitochondrial pyruvate dehydrogenase complex
Alpha-galactosidase	E2RRW9	NAGA	- Alpha-galactosidase activity<br>- Alpha-N-acetylgalactosaminidase activity<br>- Protein homodimerization activity	- Carbohydrate catabolic process<br>- Glycoside catabolic process<br>- Glycosylceramide catabolic process<br>- Oligosaccharide metabolic process	- Cytoplasm
Histone H3	E2R6K5	H3-3A	- DNA binding<br>- Protein heterodimerization activity	- Cell population proliferation<br>- Embryo implantation<br>- Mmale gonad development<br>- Multicellular organism growth<br>- Muscle cell differentiation<br>- Negative regulation of chromosome condensation<br>- Nucleus organization<br>- Oogenesis<br>- Osteoblast differentiation<br>- Pericentric heterochromatin assembly<br>- Regulation of centromere complex assembly<br>- Single fertilization<br>- Spermatid development<br>- Telomeric heterochromatin assembly	- Nucleus<br>- Chromosome

NAD(P)H quinone dehydrogenase 1	F1PBZ4	NQO1	- NAD(P)H dehydrogenase (quinone) activity<br>- Oxidoreductase activity	--	- Cytosol
Protein disulfide-isomerase	E2RD86	PDIA3	- Identical protein binding<br>- Peptide disulfide oxidoreductase activity<br>- Protein disulfide isomerase activity	- Cell redox homeostasis<br>- Cellular response to interleukin-7<br>- Positive regulation of extrinsic apoptotic signaling pathway<br>- Protein folding<br>- Response to endoplasmic reticulum stress	- Endoplasmic reticulum<br>- MHC class I peptide loading complex<br>-Extracellular space<br>- Cell surface
Lysosomal associated membrane protein 2	E2RNJ1	LAMP2	--	--	- Lysosome membrane
Tubulin beta chain	E2QYC2	TUBB6	- GTPase activity<br>- GTP binding<br>- Structural constituent of cytoskeleton	- Microtubule-based process<br>- Microtubule cytoskeleton organization<br>- Mitotic cell cycle	- Cytoskeleton
Tubulin beta chain	E2QSF4	TUBB	- GTPase activity<br>- GTP binding<br>- Structural constituent of cytoskeleton	- Microtubule-based process<br>- Microtubule cytoskeleton organization<br>- Mitotic cell cycle	- Cytoskeleton
Dihydrolipoyl dehydrogenase	F1PAR0	DLD	- Dihydrolipoyl dehydrogenase activity<br>- Flavin adenine dinucleotide binding<br>- Pyruvate dehydrogenase (NAD+) activity	- Cell redox homeostasis<br>- Gastrulation<br>- Histone succinylation<br>- Mitochondrial electron transport, NADH to ubiquinone<br>- Proteolysis<br>- Regulation of membrane potential<br>- Sperm capacitation	- Mitochondrion<br>- Nucleoplasm<br>- Acrosomal matrix<br>- Cell<br>- Cilium<br>- Oxoglutarate dehydrogenase complex<br>- Pyruvate dehydrogenase complex
Tubulin beta chain	L7N0I7	TUBB4B	- Double-stranded RNA binding<br>- GTPase activity<br>- GTP binding<br>- Structural constituent of cytoskeleton	- Microtubule-based process<br>- Microtubule cytoskeleton organization<br>- Mitotic cell cycle	- Cytoskeleton
Aspartate aminotransferase	A0A0B4J194	GOT2	- L-aspartate:2-oxoglutarate aminotransferase activity<br>- Pyridoxal phosphate binding	- Aspartate catabolic process<br>- Biosynthetic process<br>- Fatty acid transport<br>- Response to ethanol	- Mitochondrion<br>- Plasma Membrane
Valosin containing protein	E2RLQ9	VCP	- ADP binding<br>- ATPase activity<br>- ATP binding<br>- BAT3 complex binding<br>- Deubiquitinase activator activity<br>- Identical protein binding<br>- K48-linked polyubiquitin modification-dependent protein binding<br>- MHC class I protein binding<br>- Polyubiquitin modification-dependent protein binding<br>- Protein domain specific binding<br>- Protein phosphatase binding<br>- Ubiquitin protein ligase binding<br>- Ubiquitin-specific protease binding	- Activation of cysteine-type endopeptidase activity involved in apoptotic process<br>- Aggresome assembly<br>- ATP metabolic process<br>- Autophagosome maturation<br>- Cellular response to arsenite ion<br>- Cellular response to heat<br>- Double-strand break repair<br>- Endoplasmic reticulum stress-induced pre-emptive quality control<br>- Endosome to lysosome transport via multivesicular body sorting pathway<br>- ER-associated misfolded protein catabolic process<br>- Flavin adenine dinucleotide catabolic process<br>- Macroautophagy	- Cytosol<br>- Endoplasmic reticulum<br>- Nucleoplasm<br>- Nucleus<br>- ATPase complex<br>- Cytoplasm<br>- Cytoplasmic stress granule<br>- Lipid droplet<br>- Perinuclear region of cytoplasm<br>- Proteasome complex<br>- Site of double-strand break<br>- Synapse<br>- VCP-NSFL1C complex

				- Mitotic spindle disassembly<br>- NADH metabolic process<br>- Negative regulation of smoothened signaling pathway<br>- Positive regulation of ATP biosynthetic process<br>- Positive regulation of canonical Wnt signaling pathway<br>- Positive regulation of Lys63-specific deubiquitinase activity<br>- Positive regulation of mitochondrial membrane potential<br>- Positive regulation of oxidative phosphorylation<br>- Positive regulation of proteasomal ubiquitin-dependent protein catabolic process<br>- Positive regulation of protein-containing complex assembly<br>- Protein ubiquitination<br>- Regulation of aerobic respiration<br>- Retrograde protein transport, ER to cytosol<br>- Stress granule disassembly<br>- Translesion synthesis<br>- Ubiquitin-dependent ERAD pathway<br>- Viral genome replication	
Stress-70 protein, mitochondrial	E2RAU5	HSPA9	- ATPase activity<br>- ATPase activity, coupled<br>- ATP binding<br>- Heat shock protein binding<br>- Misfolded protein binding<br>- Protein folding chaperone<br>- Ubiquitin protein ligase binding<br>- Unfolded protein binding- Stress granule disassembly<br>- Translesion synthesis<br>- Ubiquitin-dependent ERAD pathway<br>- Viral genome replication	- Cellular response to unfolded protein<br>- Chaperone cofactor-dependent protein refolding<br>- Erythrocyte differentiation<br>- Iron-sulfur cluster assembly<br>- Negative regulation of erythrocyte differentiation<br>- Negative regulation of hematopoietic stem cell differentiation<br>- Protein export from nucleus<br>- Protein refolding<br>- Response to unfolded protein	- Mitochondrial nucleoid<br>- Mitochondrion<br>- Cytoplasm
Tropomyosin 4	A0A0N9JE84	TPM4	- Actin filament binding	- Actin filament organization<br>- Muscle contraction	- Cytoskeleton

Supplementary Table S5. Proteins identification and gene ontology of primary tumor control group, metastases control group, primary tumor rapamycin group and metastases rapamycin group.

Name	ID*	Gene	Gene Ontology		
			**Molecular function**	**Biological process**	**Cellular component**

Phosphoglycerate mutase	E2RT65	PGAM1	- Bisphosphoglycerate mutase activity<br>-Phosphoglycerate mutase activity<br>- Protein kinase binding	- Glycolytic process<br>- Regulation of glycolytic process<br>- Regulation of pentose-phosphate shunt<br>- Respiratory burst	- Cytosol
Dihydrolipoyl dehydrogenase	F1PAR0	DLD	- Dihydrolipoyl dehydrogenase activity<br>- Flavin adenine dinucleotide binding<br>- Pyruvate dehydrogenase (NAD+) activity	- Cell redox homeostasis<br>- Gastrulation<br>- Histone succinylation<br>- Mitochondrial electron transport, NADH to ubiquinone<br>- Proteolysis<br>- Regulation of membrane potential<br>- Sperm capacitation	- Mitochondrion<br>- Nucleoplasm<br>- Acrosomal matrix<br>- Cell<br>- Cilium<br>- Oxoglutarate dehydrogenase complex<br>- Pyruvate dehydrogenase complex
Keratin, type II	F1PTS8 (obsolete) UPI0000EB0D 72	--	--	--	--
Myotrophin	Q863Z4	MTPN	- Promotes dimerization of NF-kappa-B subunits and regulates NF-kappa-B transcription factor activity<br>- Promotes growth of cardiomyocytes, but not cardiomyocyte proliferation<br>- Promotes cardiac muscle hypertrophy<br>- Plays a role in the regulation of the growth of actin filaments<br>- Inhibits the activity of the F-actin-capping protein complex formed by the CAPZA1 and CAPZB heterodimer	- Positive regulation of cardiac muscle hypertrophy<br>- Positive regulation of cell growth<br>- Positive regulation of macromolecule biosynthetic process<br>- Positive regulation of NF-kappaB transcription factor activity<br>- Positive regulation of protein metabolic process<br>- Regulation of barbed-end actin filament capping<br>- Regulation of cell size	- Cytoskeleton<br>- Cytosol<br>- Nucleus<br>- Axon<br>- Cytoplasm<br>- Perinuclear region of cytoplasm
Tubulin beta chain	L7N0I7	TUBB4B	- Double-stranded RNA binding<br>- GTPase activity<br>- GTP binding<br>- Structural constituent of cytoskeleton	- Microtubule-based process<br>- Microtubule cytoskeleton organization<br>- Mitotic cell cycle	- Cytoskeleton
40S ribosomal protein SA	E2RJ06 (obsolete) PI0002749154	RPSA	--	--	--
Tropomyosin 4	A0A0N9JE84	TPM4	- Actin filament binding	- Actin filament organization<br>- Muscle contraction	- Cytoskeleton
Superoxide dismutase [Cu-Zn]	Q8WNN6, F1Q462	SOD1	- Copper ion binding<br>- Superoxide dismutase activity	- Reactive oxygen species metabolic process<br>- Removal of superoxide radicals	- Nucleus<br><br>- Citoplasm
Annexin	F1PXG4	ANXA4	- Calcium-dependent phospholipid binding<br>- Calcium ion binding	--	- Cytosol<br>- Plasma Membrane

					- Cytoplasm
Malate dehydrogenase 1	F1Q1R1	MDH1	- Malate dehydrogenase activity<br>- Oxidoreductase activity, acting on the CH-OH group of donors, NAD or NADP as acceptor	- Carbohydrate metabolic process<br>- Malate metabolic process	--
Nucleolin	F1Q0B0	NCL	- RNA binding	- Positive regulation of mRNA splicing, via spliceosome<br>- Regulation of RNA metabolic process	- Nucleus<br>- Spliceosomal complex<br>- Ribonucleoprotein complex
Alpha 2-HS glycoprotein	E2QUV3	AHSG	- Cysteine-type endopeptidase inhibitor activity<br>- Endopeptidase inhibitor activity	- Negative regulation of bone mineralization<br>- Negative regulation of endopeptidase activity<br>- Ossification	- Extracellular region (matrix, region, space)<br>- Golgi apparatus
Globin B2	A0A1K0FUE8	GLNB2	- Heme binding<br>- Metal ion binding<br>- Oxygen binding<br>- Oxygen carrier activity		- Cytosol (hemoglobin complex)
OBSOLETE	J9P314 (obsolete) UPI00027484BC		--	--	--
Trafficking protein particle complex subunit 2	A0A0A0MPC5	TRAPPC2	- Ion channel binding<br>- Transcription factor binding	- Endoplasmic reticulum to Golgi vesicle-mediated transport<br>- Regulation of transcription, DNA-templated<br>- Skeletal system development	- Endoplasmic reticulum<br>- - Nucleoplasm<br>- Perinuclear region of cytoplasm<br>- TRAPP complex
Eukaryotic translation elongation factor 2	F6XRY2	EEF2	- GTPase activity<br>- GTP binding<br>- Protein kinase binding<br>- Ribosome binding<br>- Translation elongation factor activity	- Hematopoietic progenitor cell differentiation<br>- Positive regulation of translation<br>- Translational elongation	- Cytosol<br>- Plasma Membrane<br>- Aggresome<br>- Polysome<br>- Ribonucleoprotein complex<br>- Synapse
Fructose-bisphosphate aldolase	F1PL63	ALDOC	- Cytoskeletal protein binding<br>- Fructose-bisphosphate aldolase activity	- Epithelial cell differentiation<br>- Fructose 1,6-bisphosphate metabolic process<br>- Glycolytic process	--
Glycyl-tRNA synthetase	F1Q332	GARS1	- ATP binding<br>- bis(5'-nucleosyl)-tetraphosphatase (asymmetrical) activity<br>- Glycine-tRNA ligase activity<br>- Identical protein binding<br>- Protein dimerization activity	- Diadenosine tetraphosphate biosynthetic process<br>- Glycyl-tRNA aminoacylation<br>- Mitochondrial glycyl-tRNA aminoacylation	- Cytosol<br>- Extracellular exosome<br>- Mitochondrion<br>- Axon<br>- Cytoplasm<br>- Secretory granule
Keratin, type I cytoskeletal 10	F1PYU9	KRT10	- Protein heterodimerization activity<br>- Structural constituent of skin epidermis	- Keratinocyte differentiation<br>- Peptide cross-linking<br>- Protein heterotetramerization	- Cytoskeleton (intermediate filament)<br>- Plasma Membrane<br>- Cytoplasm

Thioredoxin reductase 1	F1PBX0	TXNRD1	- Electron transfer activity<br>- Flavin adenine dinucleotide binding<br>- Protein disulfide oxidoreductase activity<br>- Thioredoxin-disulfide reductase activity	- Cell redox homeostasis	- Mitochondrion<br>- Cell<br>- Cytoplasm
Tropomyosin 1 alpha	A0A0N9JIB9	Tpm1	--	--	--
Uncharacterized protein	E2RKQ6	CANT1	- Scavenger receptor activity	--	- Extracellular space<br>- Membrane
NAD(P)H quinone dehydrogenase 1	F1PBZ4	NQO1	- NAD(P)H dehydrogenase (quinone) activity<br>- Oxidoreductase activity	--	- Cytosol
Annexin A2	Q6TEQ7	ANXA2	- Calcium channel activity<br>- Calcium-dependent phospholipid binding<br>- Calcium ion binding<br>- Cytoskeletal protein binding<br>- Phosphatidylinositol-4,5-bisphosphate binding<br>- Phosphatidylserine binding<br>- Phospholipase A2 inhibitor activity<br>- Protease binding<br>- Rab GTPase binding<br>- Virion binding	--	- Endosome<br>- Basement membrane<br>- Extracellular space Nucleus<br>- Plasma Membrane<br>- Cytoplasm<br>- Exocytic vesicle<br>- Vesicle
14_3_3 domain-containing protein	F1PBL1	YWHAZ	--	--	--
Uncharacterized protein	E2RJL1	LGALS1	- Carbohydrate binding	--	--
6-phosphogluconate dehydrogenase, decarboxylating	F1PE09	PGD	- NADP binding<br>- Phosphogluconate dehydrogenase (decarboxylating) activity	- D-gluconate catabolic process<br>- Pentose biosynthetic process<br>- Pentose-phosphate shunt, oxidative branch	- Cytosol
Malate dehydrogenase	F1PYG8	MDH2	- L-malate dehydrogenase activity	- Carbohydrate metabolic process<br>- Malate metabolic process<br>- Tricarboxylic acid cycle	- Mitochondrion<br>- Cytoplasm
Cytochrome c	P00011	CYCS	- Electron transporter, transferring electrons from CoQH2-cytochrome c reductase complex and cytochrome c oxidase complex activity<br>- Heme binding<br>- Metal ion binding	- Apoptotic process<br>- Mitochondrial electron transport, cytochrome c to oxygen<br>- Mitochondrial electron transport, ubiquinol to cytochrome c<br>- Positive regulation of cysteine-type endopeptidase activity involved in apoptotic process	- Cytosol<br>- Mitochondrial intermembrane space<br>- Nucleus<br>- Respirasome
Dihydropyrimidinase like 2	F1P9U4	DPYSL2	- Hydrolase activity, acting on carbon-nitrogen (but not peptide) bonds, in cyclic amides<br>- Microtubule binding<br>- Protein kinase binding	- Axon guidance<br>- Brain development<br>- Cytoskeleton organization<br>- Regulation of axon extension	- Cytosol<br>- Plasma Membrane<br>- Axon<br>- Dendrite<br>- Neuronal cell body

Supplementary Table S6. Mass spectrometry data of mammary gland tumor cells from dogs.

Symbol	Score	Mass	Matches	Sequences	Name
E2RJ06	182	35182	2 (2)	1 (1)	40S ribosomal protein SA OS=Canis lupus familiaris OX=9615 GN=RPSA PE=3 SV=2
F1P797	70	65257	1 (1)	1 (1)	5-aminoimidazole-4-carboxamide ribonucleotide formyltransferase/IMP cyclohydrolase OS=Canis lupus familiaris OX=9615 GN=ATIC PE=3 SV=2
F1PE09	162	53575	5 (3)	3 (2)	6-phosphogluconate dehydrogenase, decarboxylating OS=Canis lupus familiaris OX=9615 GN=PGD PE=3 SV=2
E2QUU5	1168	61128	23 (20)	6 (6)	60 kDa heat shock protein, mitochondrial OS=Canis lupus familiaris OX=9615 GN=HSPD1 PE=3 SV=2
F1PC58	367	44246	8 (7)	3 (3)	Acetyl-CoA acetyltransferase 1 OS=Canis lupus familiaris OX=9615 GN=ACAT1 PE=3 SV=2
F1PQ43	40	26534	1 (1)	1 (1)	Acidic leucine-rich nuclear phosphoprotein 32 family member A OS=Canis lupus familiaris OX=9615 GN=ANP32A PE=4 SV=2
E2RCY8	215	86409	6 (4)	3 (3)	Aconitate hydratase, mitochondrial OS=Canis lupus familiaris OX=9615 GN=ACO2 PE=3 SV=1
Z4YHI2, F1PQL8	6213	42365	177 (142)	25 (21)	Actin gamma 1 OS=Canis lupus familiaris OX=9615 GN=ACTG1 PE=3 SV=1
F1PVC1	953	37588	64 (32)	10 (8)	Actin, alpha 1, skeletal muscle OS=Canis lupus familiaris OX=9615 GN=ACTA1 PE=3 SV=2
F2Z4N7	1356	42334	39 (36)	11 (10)	Actin, alpha, cardiac muscle 1 OS=Canis lupus familiaris OX=9615 GN=ACTC1 PE=3 SV=1
F1PAC7	235	12303	5 (5)	3 (3)	Acyl-CoA-binding protein OS=Canis lupus familiaris OX=9615 GN=DBI PE=4 SV=2
E2QXS7	268	48345	7 (7)	6 (6)	Adenosylhomocysteinase OS=Canis lupus familiaris OX=9615 GN=AHCY PE=3 SV=2
E2RE39	56	26781	1 (1)	1 (1)	Adenylate kinase 2, mitochondrial OS=Canis lupus familiaris OX=9615 GN=AK2 PE=3 SV=2
J9P969	164	533922	8 (4)	3 (2)	AHNAK nucleoprotein OS=Canis lupus familiaris OX=9615 GN=AHNAK PE=4 SV=1
F1P7C2	37	107450	2 (2)	1 (1)	Alanyl-tRNA synthetase OS=Canis lupus familiaris OX=9615 GN=AARS PE=3 SV=2
E2RQ99	94	59116	5 (3)	3 (2)	Aldehyde dehydrogenase 7 family member A1 OS=Canis lupus familiaris OX=9615 GN=ALDH7A1 PE=3 SV=1
F1PK43	117	36895	6 (4)	2 (2)	Aldo-keto reductase family 1 member A1 OS=Canis lupus familiaris OX=9615 GN=AKR1A1 PE=4 SV=2
E2QUV3	56	40021	4 (3)	2 (2)	Alpha 2-HS glycoprotein OS=Canis lupus familiaris OX=9615 GN=AHSG PE=4 SV=2
L7N0N6	53	51884	1 (1)	1 (1)	Alpha-amylase OS=Canis lupus familiaris OX=9615 GN=LOC479922 PE=3 SV=1
F1PXN2	66	70491	4 (2)	2 (1)	Alpha-fetoprotein OS=Canis lupus familiaris OX=9615 GN=AFP PE=4 SV=1

E2RRW9	329	46832	7 (7)	5 (5)	Alpha-galactosidase OS=Canis lupus familiaris OX=9615 GN=NAGA PE=3 SV=2
F1PKB2	340	113563	5 (5)	3 (3)	Alpha-mannosidase OS=Canis lupus familiaris OX=9615 GN=MAN2B1 PE=3 SV=2
Q6TEQ7	591	38915	12 (12)	8 (8)	Annexin A2 OS=Canis lupus familiaris OX=9615 GN=ANXA2 PE=1 SV=1
F1P6B7	1263	38887	23 (21)	8 (8)	Annexin OS=Canis lupus familiaris OX=9615 GN=ANXA1 PE=3 SV=1
F1PXG4	86	36144	2 (2)	1 (1)	Annexin OS=Canis lupus familiaris OX=9615 GN=ANXA4 PE=3 SV=2
E2RQ14	796	35978	18 (17)	6 (6)	Annexin OS=Canis lupus familiaris OX=9615 GN=ANXA5 PE=2 SV=2
E2RCI8	186	76520	2 (2)	1 (1)	Annexin OS=Canis lupus familiaris OX=9615 GN=ANXA6 PE=3 SV=2
E2R0S6	266	42874	3 (3)	1 (1)	Annexin OS=Canis lupus familiaris OX=9615 GN=ANXA8L1 PE=3 SV=2
E2RKW9	155	39321	2 (2)	1 (1)	Annexin OS=Canis lupus familiaris OX=9615 PE=3 SV=2
A0A0B4J194	214	47809	4 (4)	3 (3)	Aspartate aminotransferase OS=Canis lupus familiaris OX=9615 GN=GOT2 PE=4 SV=1
F1PZW1	28	30631	2 (1)	1 (1)	Aspartate dehydrogenase domain containing OS=Canis lupus familiaris OX=9615 GN=ASPDH PE=4 SV=2
E2QYN4	42	12163	1 (1)	1 (1)	ATP synthase inhibitory factor subunit 1 OS=Canis lupus familiaris OX=9615 GN=ATP5IF1 PE=4 SV=1
A0A1B1X469	767	59805	11 (11)	4 (4)	ATP synthase subunit alpha OS=Canis lupus familiaris OX=9615 PE=2 SV=1
Q0QEN2	268	34665	4 (4)	2 (2)	ATP synthase subunit beta (Fragment) OS=Canis lupus familiaris OX=9615 GN=ATP5B PE=2 SV=1
F1PDB4	807	56250	16 (15)	7 (6)	ATP synthase subunit beta OS=Canis lupus familiaris OX=9615 GN=ATP5F1B PE=2 SV=1
A0A1B1X461	28	33089	3 (1)	2 (1)	ATP synthase subunit gamma OS=Canis lupus familiaris OX=9615 PE=2 SV=1
E2QXA4	27	305403	1 (1)	1 (1)	ATR serine/threonine kinase OS=Canis lupus familiaris OX=9615 GN=ATR PE=3 SV=2
F1PQM0	40	270356	7 (3)	2 (1)	BAH domain and coiled-coil containing 1 OS=Canis lupus familiaris OX=9615 GN=BAHCC1 PE=4 SV=2
F1PYK4	27	286093	2 (1)	1 (1)	Bassoon presynaptic cytomatrix protein OS=Canis lupus familiaris OX=9615 GN=BSN PE=4 SV=2
F1PDK3	118	66904	2 (2)	1 (1)	Beta-galactosidase OS=Canis lupus familiaris OX=9615 GN=GLB1 PE=3 SV=2
K0J5K2	232	52131	8 (7)	4 (4)	Beta-N-acetylhexosaminidase beta subunit, exons 2-14 (Fragment) OS=Canis lupus familiaris OX=9615 GN=HEXB PE=4 SV=1
E2QVU9	341	22236	6 (5)	3 (2)	Biliverdin reductase B OS=Canis lupus familiaris OX=9615 GN=BLVRB PE=4 SV=1
F1PY28	269	43366	3 (3)	1 (1)	Branched-chain-amino-acid aminotransferase OS=Canis lupus familiaris OX=9615 GN=BCAT1 PE=3 SV=2
E2R7S1	19	135549	3 (1)	1 (1)	Calcium-transporting ATPase OS=Canis lupus familiaris OX=9615 GN=ATP2B1 PE=3 SV=2

F1Q0J3	164	83054	2 (2)	1 (1)	Caldesmon 1 OS=Canis lupus familiaris OX=9615 GN=CALD1 PE=4 SV=2
F1PX12	28	79101	1 (1)	1 (1)	Calpastatin OS=Canis lupus familiaris OX=9615 GN=CAST PE=4 SV=2
F6UYJ9	962	47278	17 (16)	6 (6)	Calreticulin OS=Canis lupus familiaris OX=9615 GN=CALR PE=3 SV=1
E2RN38	141	37169	6 (5)	3 (2)	Calumenin OS=Canis lupus familiaris OX=9615 GN=CALU PE=4 SV=1
F1PSP6	115	51303	2 (2)	1 (1)	Carboxypeptidase OS=Canis lupus familiaris OX=9615 GN=SCPEP1 PE=3 SV=2
E2R6Q7	626	38687	8 (8)	2 (2)	Cathepsin B OS=Canis lupus familiaris OX=9615 GN=CTSB PE=3 SV=1
F1PIF2	183	29897	4 (4)	3 (3)	Cathepsin Z OS=Canis lupus familiaris OX=9615 GN=CTSZ PE=3 SV=2
E2QU88	20	64223	2 (1)	1 (1)	Checkpoint kinase 2 OS=Canis lupus familiaris OX=9615 GN=CHEK2 PE=4 SV=2
E2REQ7	25	11170	1 (1)	1 (1)	Chromosome 11 open reading frame 94 OS=Canis lupus familiaris OX=9615 GN=C18H11orf94 PE=4 SV=1
F1PF60	13	68352	3 (1)	1 (1)	Chromosome 27 C12orf40 homolog OS=Canis lupus familiaris OX=9615 GN=C27H12orf40 PE=4 SV=2
Q0QEK9	44	23719	1 (1)	1 (1)	Citrate synthase (Fragment) OS=Canis lupus familiaris OX=9615 GN=CS PE=2 SV=1
F1PHQ0	25	193333	1 (1)	1 (1)	Clathrin heavy chain OS=Canis lupus familiaris OX=9615 GN=CLTC PE=3 SV=2
E2QU79	26	67466	1 (1)	1 (1)	Coenzyme A synthase OS=Canis lupus familiaris OX=9615 GN=COASY PE=3 SV=2
E2QUS9	264	10922	7 (7)	5 (5)	Coenzyme Q10B OS=Canis lupus familiaris OX=9615 GN=COQ10B PE=3 SV=2
F1PQN5	536	18716	6 (5)	3 (2)	Cofilin 1 OS=Canis lupus familiaris OX=9615 GN=CFL1 PE=3 SV=2
F1Q3I5	36	140042	1 (1)	1 (1)	Collagen alpha-1(I) chain OS=Canis lupus familiaris OX=9615 GN=COL1A1 PE=4 SV=2
F1PG69	128	139948	6 (3)	3 (2)	Collagen type III alpha 1 chain OS=Canis lupus familiaris OX=9615 GN=COL3A1 PE=4 SV=2
P05124	992	42960	18 (17)	6 (5)	Creatine kinase B-type OS=Canis lupus familiaris OX=9615 GN=CKB PE=1 SV=1
F1PFQ4	37	161390	1 (1)	1 (1)	Crystallin beta-gamma domain containing 1 OS=Canis lupus familiaris OX=9615 GN=CRYBG1 PE=3 SV=2
E2RCL5	45	33832	1 (1)	1 (1)	Crystallin mu OS=Canis lupus familiaris OX=9615 GN=CRYM PE=4 SV=1
F1PS73	140	9014	3 (2)	2 (1)	Cystatin B OS=Canis lupus familiaris OX=9615 GN=CSTB PE=4 SV=2
E2R003	29	121282	6 (1)	2 (1)	Cysteine rich transmembrane BMP regulator 1 OS=Canis lupus familiaris OX=9615 GN=CRIM1 PE=4 SV=2
P00011	190	11739	7 (6)	3 (3)	Cytochrome c OS=Canis lupus familiaris OX=9615 GN=CYCS PE=1 SV=2
F1PAR0	146	54789	4 (4)	2 (2)	Dihydrolipoyl dehydrogenase OS=Canis lupus familiaris OX=9615 GN=DLD PE=3 SV=2

A0A0E3V3B7	20	36906	3 (1)	1 (1)	Dihydroorotate dehydrogenase (quinone) OS=Pasteurella multocida subsp. multocida OH4807 OX=1304873 GN=pyrD PE=3 SV=1
F1P9U4	164	74058	2 (2)	1 (1)	Dihydropyrimidinase like 2 OS=Canis lupus familiaris OX=9615 GN=DPYSL2 PE=4 SV=2
F1Q3Y2	139	62236	2 (2)	1 (1)	Dihydropyrimidinase like 3 OS=Canis lupus familiaris OX=9615 GN=DPYSL3 PE=4 SV=2
F6XH37	66	55199	2 (2)	1 (1)	Dipeptidyl peptidase 7 OS=Canis lupus familiaris OX=9615 GN=DPP7 PE=4 SV=1
E2RAE2	194	35318	4 (4)	2 (2)	Electron transfer flavoprotein alpha subunit OS=Canis lupus familiaris OX=9615 GN=ETFA PE=4 SV=2
Q7M3A6	130	12536	5 (4)	3 (2)	Endogenous anti-morphine peptide OS=Canis lupus familiaris OX=9615 PE=1 SV=1
J9P4L2	70	41870	2 (2)	1 (1)	Endoplasmic reticulum protein 29 OS=Canis lupus familiaris OX=9615 GN=ERP29 PE=4 SV=1
F1PCH3	1276	47175	42 (29)	14 (12)	Enolase 1 OS=Canis lupus familiaris OX=9615 GN=ENO1 PE=3 SV=2
E2R921	34	38969	1 (1)	1 (1)	Enoyl-CoA hydratase 1 OS=Canis lupus familiaris OX=9615 GN=ECH1 PE=3 SV=2
F6XRY2	31	96248	1 (1)	1 (1)	Eukaryotic translation elongation factor 2 OS=Canis lupus familiaris OX=9615 GN=EEF2 PE=4 SV=1
F1Q264	142	17049	4 (2)	3 (2)	Eukaryotic translation initiation factor 5A OS=Canis lupus familiaris OX=9615 GN=EIF5A PE=3 SV=2
F1PYU3	176	55296	2 (2)	1 (1)	Fascin OS=Canis lupus familiaris OX=9615 GN=FSCN1 PE=3 SV=2
E2R507	83	14853	2 (2)	1 (1)	Fatty acid binding protein 3 OS=Canis lupus familiaris OX=9615 GN=FABP3 PE=3 SV=1
E2R9U9	36	118254	1 (1)	1 (1)	FERM, ARH/RhoGEF and pleckstrin domain protein 2 OS=Canis lupus familiaris OX=9615 GN=FARP2 PE=4 SV=2
E2RMZ3	401	20097	6 (6)	3 (3)	Ferritin OS=Canis lupus familiaris OX=9615 PE=3 SV=2
Q28275	166	58235	2 (2)	1 (1)	Fibronectin (Fragment) OS=Canis lupus familiaris OX=9615 GN=FN1 PE=2 SV=2
E2RIM4	64	71647	2 (2)	1 (1)	Frizzled class receptor 2 OS=Canis lupus familiaris OX=9615 GN=FZD2 PE=3 SV=2
F1PBT3	237	76161	7 (7)	3 (3)	Fructose-bisphosphate aldolase OS=Canis lupus familiaris OX=9615 GN=ALDOA PE=3 SV=2
F1PL63	129	39716	3 (1)	3 (1)	Fructose-bisphosphate aldolase OS=Canis lupus familiaris OX=9615 GN=ALDOC PE=3 SV=1
E2RGR9	228	54588	3 (3)	2 (2)	Fumarate hydratase OS=Canis lupus familiaris OX=9615 GN=FH PE=3 SV=1
E2RKQ6	249	63197	5 (5)	3 (3)	Galectin 3 binding protein OS=Canis lupus familiaris OX=9615 GN=LGALS3BP PE=4 SV=1
E2RJL1	66	15122	2 (2)	2 (2)	Galectin OS=Canis lupus familiaris OX=9615 GN=LGALS1 PE=2 SV=1
E5Q8W5	75	29544	2 (2)	1 (1)	Galectin OS=Canis lupus familiaris OX=9615 GN=LGALS3 PE=2 SV=1
E2QUT9	67	35876	2 (2)	2 (2)	Gamma-glutamyl hydrolase OS=Canis lupus familiaris OX=9615 GN=GGH PE=4 SV=2

A0A1K0FUE8	53	16493	2 (2)	1 (1)	Globin B2 OS=Canis lupus familiaris OX=9615 GN=GLNB2 PE=3 SV=1
E2R2C3	602	63026	18 (13)	6 (6)	Glucose-6-phosphate isomerase OS=Canis lupus familiaris OX=9615 GN=GPI PE=3 SV=1
F1Q4J0	107	106768	5 (3)	2 (2)	Glucosidase II alpha subunit OS=Canis lupus familiaris OX=9615 GN=GANAB PE=3 SV=2
Q9TQQ6	58	3551	3 (1)	2 (1)	Glutathione S-transferase PI isozyme YD1-2YD1-2(IV-HB) (Fragment) OS=Canis lupus familiaris OX=9615 PE=1 SV=1
F1PTZ9, Q28259	1643	35682	45 (32)	11 (10)	Glyceraldehyde-3-phosphate dehydrogenase OS=Canis lupus familiaris OX=9615 GN=GAPDH PE=3 SV=2
F1P7C9	904	36075	16 (13)	8 (7)	Glyceraldehyde-3-phosphate dehydrogenase OS=Canis lupus familiaris OX=9615 GN=LOC100688969 PE=3 SV=2
E2QUR6	45	64962	1 (1)	1 (1)	Glycoprotein nmb OS=Canis lupus familiaris OX=9615 GN=GPNMB PE=4 SV=1
F1Q332	69	89854	1 (1)	1 (1)	Glycyl-tRNA synthetase OS=Canis lupus familiaris OX=9615 GN=GARS PE=4 SV=2
F1P601	25	71279	1 (1)	1 (1)	Guanylate cyclase soluble subunit beta-1 OS=Canis lupus familiaris OX=9615 GN=GUCY1B1 PE=3 SV=2
A0A097HUC9	49	22826	1 (1)	1 (1)	Heat shock protein 27 (Fragment) OS=Canis lupus familiaris OX=9615 GN=HSP27 PE=2 SV=1
F1PYE3	212	22809	5 (5)	3 (3)	Heat shock protein 27 kDa beta-1 OS=Canis lupus familiaris OX=9615 GN=HSPB1 PE=3 SV=1
F1PGY1	81	73391	3 (1)	3 (1)	Heat shock protein 90 alpha family class A member 1 OS=Canis lupus familiaris OX=9615 GN=HSP90AA1 PE=4 SV=2
E2QY26	33	75712	1 (1)	1 (1)	Heat shock protein family A (Hsp70) member 12B OS=Canis lupus familiaris OX=9615 GN=HSPA12B PE=4 SV=1
F1PIC7	1585	72352	43 (37)	21 (18)	Heat shock protein family A (Hsp70) member 5 OS=Canis lupus familiaris OX=9615 GN=HSPA5 PE=3 SV=2
F1PWM7	90	17382	2 (2)	1 (1)	Heat shock protein family B (small) member 6 OS=Canis lupus familiaris OX=9615 GN=HSPB6 PE=3 SV=2
F1PA19	141	29902	2 (2)	1 (1)	Heterogeneous nuclear ribonucleoprotein A/B OS=Canis lupus familiaris OX=9615 GN=HNRNPAB PE=4 SV=2
E2RNB0	177	15119	8 (7)	3 (3)	Histone H2A OS=Canis lupus familiaris OX=9615 GN=H2AFX PE=3 SV=1
F1P790	406	14143	7 (7)	2 (2)	Histone H2A OS=Canis lupus familiaris OX=9615 GN=HIST3H2A PE=3 SV=2
E2RNW2, F1P782	783	14707	13 (11)	4 (4)	Histone H2B OS=Canis lupus familiaris OX=9615 PE=3 SV=1
E2R6K5	70	15376	4 (4)	2 (2)	Histone H3 OS=Canis lupus familiaris OX=9615 GN=LOC475916 PE=3 SV=1
J9P7X1	73	15436	3 (2)	3 (2)	Histone H3 OS=Canis lupus familiaris OX=9615 GN=LOC483172 PE=3 SV=1
F2Z4N2	272	11360	7 (7)	5 (5)	Histone H4 OS=Canis lupus familiaris OX=9615 GN=LOC100856216 PE=3 SV=1
E2RMC1	99	44290	1 (1)	1 (1)	Hsc70-interacting protein OS=Canis lupus familiaris OX=9615 GN=ST13 PE=4 SV=2

F1PBL9	34	269381	2 (1)	2 (1)	Human immunodeficiency virus type I enhancer binding protein 2 OS=Canis lupus familiaris OX=9615 GN=HIVEP2 PE=4 SV=1
F6XHT8	64	31927	5 (2)	3 (1)	Hydroxyacyl-CoA dehydrogenase OS=Canis lupus familiaris OX=9615 GN=HADH PE=4 SV=1
E2R7R1	102	19277	2 (2)	1 (1)	ISG15 ubiquitin-like modifier OS=Canis lupus familiaris OX=9615 GN=ISG15 PE=4 SV=2
Q0QEQ8	86	41074	2 (2)	1 (1)	Isocitrate dehydrogenase 1 (Fragment) OS=Canis lupus familiaris OX=9615 GN=IDH1 PE=2 SV=1
E2R8Z5	191	62919	6 (3)	3 (1)	Keratin 5 OS=Canis lupus familiaris OX=9615 GN=KRT5 PE=3 SV=2
F1Q0N7	93	65274	2 (2)	1 (1)	Keratin 9 OS=Canis lupus familiaris OX=9615 GN=KRT9 PE=3 SV=1
F1PYU9	324	57821	11 (11)	7 (7)	Keratin, type I cytoskeletal 10 OS=Canis lupus familiaris OX=9615 GN=KRT10 PE=3 SV=2
F1PTY1	766	63846	17 (17)	7 (7)	Keratin, type II cytoskeletal 1 OS=Canis lupus familiaris OX=9615 GN=KRT1 PE=3 SV=1
F1PTX4	92	65016	1 (1)	1 (1)	Keratin, type II cytoskeletal 2 epidermal OS=Canis lupus familiaris OX=9615 GN=KRT2 PE=3 SV=2
F1PVW0	1286	40058	28 (24)	11 (9)	L-lactate dehydrogenase OS=Canis lupus familiaris OX=9615 GN=LDHA PE=3 SV=2
F1PIB3, E2R761, J9NT18	365	39631	8 (6)	5 (3)	L-lactate dehydrogenase OS=Canis lupus familiaris OX=9615 GN=LDHB PE=3 SV=2
J9NRV6	64	20982	2 (1)	1 (1)	Lactoylglutathione lyase OS=Canis lupus familiaris OX=9615 GN=GLO1 PE=3 SV=1
E2RNJ1	69	45677	5 (3)	2 (1)	Lysosomal associated membrane protein 2 OS=Canis lupus familiaris OX=9615 GN=LAMP2 PE=3 SV=2
Q0QF34	716	31526	12 (12)	7 (7)	Malate dehydrogenase (Fragment) OS=Canis lupus familiaris OX=9615 GN=MDH2 PE=2 SV=1
F1Q1R1	133	30492	2 (2)	2 (2)	Malate dehydrogenase OS=Canis lupus familiaris OX=9615 GN=MDH1 PE=3 SV=2
F1PYG8	1027	35883	20 (19)	10 (9)	Malate dehydrogenase OS=Canis lupus familiaris OX=9615 GN=MDH2 PE=3 SV=1
F1Q2T9	79	24776	3 (3)	2 (2)	Malic enzyme 1 OS=Canis lupus familiaris OX=9615 GN=ME1 PE=4 SV=1
J9NZX7	233	55623	3 (3)	3 (3)	Malic enzyme OS=Canis lupus familiaris OX=9615 GN=ME1 PE=3 SV=1
F1P8E4	30	38848	36 (1)	1 (1)	Mitochondrial ribosomal protein L39 OS=Canis lupus familiaris OX=9615 GN=MRPL39 PE=4 SV=2
E2REQ0	18	75521	3 (2)	1 (1)	Mitogen-activated protein kinase 8 interacting protein 1 OS=Canis lupus familiaris OX=9615 GN=MAPK8IP1 PE=4 SV=2
E2R7F1	82	67981	2 (2)	1 (1)	Moesin OS=Canis lupus familiaris OX=9615 GN=MSN PE=4 SV=2
E2R856	39	18928	1 (1)	1 (1)	Myeloid derived growth factor OS=Canis lupus familiaris OX=9615 GN=MYDGF PE=4 SV=2
Q863Z4	177	13058	2 (2)	1 (1)	Myotrophin OS=Canis lupus familiaris OX=9615 GN=MTPN PE=3 SV=3

F1P6L7	106	56144	2 (2)	1 (1)	N-acetylglucosamine-6-sulfatase OS=Canis lupus familiaris OX=9615 GN=GNS PE=3 SV=2
F1PTW7	138	47638	3 (3)	1 (1)	N-sulfoglucosamine sulfohydrolase OS=Canis lupus familiaris OX=9615 GN=SGSH PE=4 SV=2
F1PBZ4	192	30832	2 (2)	1 (1)	NAD(P)H quinone dehydrogenase 1 OS=Canis lupus familiaris OX=9615 GN=NQO1 PE=4 SV=1
F1PNP2	32	125656	1 (1)	1 (1)	Neurofilament heavy OS=Canis lupus familiaris OX=9615 GN=NEFH PE=3 SV=2
F1PAR9	90	16976	4 (2)	2 (1)	NPC intracellular cholesterol transporter 2 OS=Canis lupus familiaris OX=9615 GN=NPC2 PE=4 SV=2
F1PLT4	21	49177	2 (1)	1 (1)	Nuclear autoantigenic sperm protein OS=Canis lupus familiaris OX=9615 GN=NASP PE=4 SV=2
F1Q0B0	426	77493	7 (7)	3 (3)	Nucleolin OS=Canis lupus familiaris OX=9615 GN=NCL PE=4 SV=2
E2RC20, Q50KA9	583	32882	16 (14)	8 (6)	Nucleoside diphosphate kinase A OS=Canis lupus familiaris OX=9615 GN=NME1 PE=3 SV=2
J9PAG4	49	33056	4 (4)	1 (1)	Nudix hydrolase 19 OS=Canis lupus familiaris OX=9615 GN=NUDT19 PE=4 SV=1
F1PI87	149	45288	3 (3)	1 (1)	Obg-like ATPase 1 OS=Canis lupus familiaris OX=9615 GN=OLA1 PE=3 SV=2
E2QS13	306	20170	6 (5)	3 (3)	Parkinsonism associated deglycase OS=Canis lupus familiaris OX=9615 GN=PARK7 PE=4 SV=1
F1PLV2	506	23818	12 (10)	7 (6)	Peptidyl-prolyl cis-trans isomerase OS=Canis lupus familiaris OX=9615 GN=PPIB PE=3 SV=2
J9NV93, F1PK62	1504	16846	23 (23)	7 (7)	Peptidyl-prolyl cis-trans isomerase OS=Canis lupus familiaris OX=9615 PE=3 SV=1
F1PWN3	180	9124	2 (2)	1 (1)	Peptidylprolyl isomerase OS=Canis lupus familiaris OX=9615 GN=FKBP1A PE=4 SV=2
F1PCG4	271	22112	7 (5)	3 (3)	Peroxiredoxin 2 OS=Canis lupus familiaris OX=9615 GN=PRDX2 PE=4 SV=2
F1PC59	325	24713	8 (8)	3 (3)	Peroxiredoxin 6 OS=Canis lupus familiaris OX=9615 GN=PRDX6 PE=4 SV=1
Q3YIX4, F1P699	928	21080	14 (14)	7 (7)	Phosphatidylethanolamine-binding protein 1 OS=Canis lupus familiaris OX=9615 GN=PEBP1 PE=1 SV=1
F1PUL4	90	64930	2 (2)	2 (2)	Phosphoglucomutase 1 OS=Canis lupus familiaris OX=9615 GN=PGM1 PE=3 SV=2
E2RRC9	770	44890	12 (12)	5 (5)	Phosphoglycerate kinase OS=Canis lupus familiaris OX=9615 GN=PGK1 PE=3 SV=1
E2RT65	267	28918	5 (5)	1 (1)	Phosphoglycerate mutase OS=Canis lupus familiaris OX=9615 GN=PGAM1 PE=3 SV=2
E2RT75	24	135262	4 (1)	2 (1)	Phosphoinositide phospholipase C OS=Canis lupus familiaris OX=9615 GN=PLCB2 PE=4 SV=1
F1PFI3	38	20634	2 (2)	1 (1)	Phospholipase C beta 3 OS=Canis lupus familiaris OX=9615 GN=PLCB3 PE=4 SV=2
E2RDN1	181	45641	5 (4)	4 (4)	Phosphoserine aminotransferase OS=Canis lupus familiaris OX=9615 GN=PSAT1 PE=3 SV=2

F6UKT8	94	32243	3 (2)	2 (1)	Pirin OS=Canis lupus familiaris OX=9615 GN=PIR PE=3 SV=1
F1PWW9	31	148718	1 (1)	1 (1)	Pleckstrin homology and RhoGEF domain containing G3 OS=Canis lupus familiaris OX=9615 GN=PLEKHG3 PE=4 SV=2
F1PA33	54	93690	3 (3)	1 (1)	Potassium calcium-activated channel subfamily N member 2 OS=Canis lupus familiaris OX=9615 GN=KCNN2 PE=4 SV=2
F1Q3Y0	256	10732	6 (6)	2 (2)	Profilin OS=Canis lupus familiaris OX=9615 GN=PFN1 PE=3 SV=1
E2R0D6	93	29102	2 (2)	2 (2)	Proliferating cell nuclear antigen OS=Canis lupus familiaris OX=9615 GN=PCNA PE=3 SV=1
F1PTP6	74	61009	2 (2)	1 (1)	Prolyl 4-hydroxylase subunit alpha 2 OS=Canis lupus familiaris OX=9615 GN=P4HA2 PE=4 SV=2
F1PHW5	235	80358	3 (3)	1 (1)	Prolyl endopeptidase OS=Canis lupus familiaris OX=9615 GN=PREP PE=4 SV=2
E2R002	325	35937	8 (7)	5 (4)	Prostaglandin reductase 1 OS=Canis lupus familiaris OX=9615 GN=PTGR1 PE=4 SV=2
E2R4H4	29	20136	1 (1)	1 (1)	Proteasome endopeptidase complex OS=Canis lupus familiaris OX=9615 GN=PSMA5 PE=3 SV=2
F1PUB5	71	24737	1 (1)	1 (1)	Proteasome endopeptidase complex OS=Canis lupus familiaris OX=9615 GN=PSMA7 PE=3 SV=2
E2RKR4	107	28657	3 (2)	1 (1)	Proteasome subunit alpha type OS=Canis lupus familiaris OX=9615 GN=PSMA3 PE=3 SV=1
E2RMN2	124	27824	2 (2)	1 (1)	Proteasome subunit alpha type OS=Canis lupus familiaris OX=9615 GN=PSMA6 PE=3 SV=1
E2R1C3	107	27762	1 (1)	1 (1)	Proteasome subunit alpha type OS=Canis lupus familiaris OX=9615 PE=3 SV=2
F1PF02	107	20624	1 (1)	1 (1)	Proteasome subunit beta type OS=Canis lupus familiaris OX=9615 GN=PSMB1 PE=3 SV=2
E2QX17	40	23249	2 (1)	1 (1)	Proteasome subunit beta type OS=Canis lupus familiaris OX=9615 GN=PSMB3 PE=3 SV=1
E2R3R2	83	28687	1 (1)	1 (1)	Proteasome subunit beta type OS=Canis lupus familiaris OX=9615 GN=PSMB5 PE=3 SV=1
E2R0B6	70	25686	1 (1)	1 (1)	Proteasome subunit beta type OS=Canis lupus familiaris OX=9615 GN=PSMB6 PE=3 SV=1
E2R7L1	97	72693	4 (3)	2 (2)	Protein disulfide-isomerase A4 OS=Canis lupus familiaris OX=9615 GN=PDIA4 PE=3 SV=2
F1PL97	299	57780	5 (5)	3 (3)	Protein disulfide-isomerase OS=Canis lupus familiaris OX=9615 GN=P4HB PE=3 SV=2
E2RD86	450	57171	12 (12)	9 (9)	Protein disulfide-isomerase OS=Canis lupus familiaris OX=9615 GN=PDIA3 PE=3 SV=1
E2R8U0	27	83268	1 (1)	1 (1)	Protein phosphatase 1 regulatory subunit 3F OS=Canis lupus familiaris OX=9615 GN=PPP1R3F PE=4 SV=1
F1PFF2	273	11231	4 (4)	2 (2)	Protein S100 OS=Canis lupus familiaris OX=9615 GN=S100A11 PE=3 SV=2
F1PQM1	64	32566	3 (2)	2 (1)	Purine nucleoside phosphorylase OS=Canis lupus familiaris OX=9615 GN=PNP PE=3 SV=2
F1PHR2	1368	60190	30 (27)	11 (10)	Pyruvate kinase OS=Canis lupus familiaris OX=9615 GN=PKM PE=3 SV=2

F1P8L7	351	49285	9 (6)	6 (4)	Rab GDP dissociation inhibitor OS=Canis lupus familiaris OX=9615 GN=GDI2 PE=3 SV=1
F1PWQ0	349	51044	5 (5)	3 (3)	Rab GDP dissociation inhibitor OS=Canis lupus familiaris OX=9615 GN=PLXNA3 PE=3 SV=2
E2R4A5	95	43027	2 (2)	1 (1)	RAD23 homolog B, nucleotide excision repair protein OS=Canis lupus familiaris OX=9615 GN=RAD23B PE=4 SV=2
E2RHK9	134	24251	2 (2)	1 (1)	RAN binding protein 1 OS=Canis lupus familiaris OX=9615 GN=RANBP1 PE=4 SV=2
F1PLR0	301	35511	3 (3)	1 (1)	Receptor for activated C kinase 1 OS=Canis lupus familiaris OX=9615 GN=RACK1 PE=4 SV=2
F1Q1T5	47	13352	1 (1)	1 (1)	Ribosomal protein S15 OS=Canis lupus familiaris OX=9615 GN=RPS15 PE=3 SV=1
E2RIQ8	176	23033	2 (2)	1 (1)	Ribosomal protein S5 OS=Canis lupus familiaris OX=9615 GN=RPS5 PE=3 SV=1
E2R8Z4	24	128919	5 (1)	2 (1)	RNA binding motif protein 6 OS=Canis lupus familiaris OX=9615 GN=RBM6 PE=4 SV=1
F1P9L1	120	31813	2 (2)	1 (1)	S-methyl-5'-thioadenosine phosphorylase OS=Canis lupus familiaris OX=9615 GN=MTAP PE=3 SV=2
E2RMA3	188	35390	3 (2)	2 (1)	Secreted protein acidic and cysteine rich OS=Canis lupus familiaris OX=9615 GN=SPARC PE=4 SV=1
F1P979	25	131228	4 (1)	1 (1)	SEL1L family member 3 OS=Canis lupus familiaris OX=9615 GN=SEL1L3 PE=4 SV=2
E2R4L7	168	59617	2 (2)	1 (1)	Serine hydroxymethyltransferase OS=Canis lupus familiaris OX=9615 GN=SHMT2 PE=3 SV=2
E2RHY7	456	46724	6 (6)	3 (3)	Serpin family H member 1 OS=Canis lupus familiaris OX=9615 GN=SERPINH1 PE=1 SV=1
F2Z4Q6	695	70558	15 (11)	5 (3)	Serum albumin OS=Canis lupus familiaris OX=9615 GN=ALB PE=4 SV=1
E2RH09	135	46842	2 (2)	1 (1)	Sjogren syndrome antigen B OS=Canis lupus familiaris OX=9615 GN=SSB PE=4 SV=2
E2RIW7	32	13273	2 (1)	1 (1)	Small nuclear ribonucleoprotein D1 polypeptide OS=Canis lupus familiaris OX=9615 GN=SNRPD1 PE=4 SV=1
E2RGN5	154	10921	2 (2)	1 (1)	Small ubiquitin-related modifier OS=Canis lupus familiaris OX=9615 GN=SUMO2 PE=3 SV=1
F1PKH1	23	117187	2 (1)	1 (1)	Solute carrier family 12 member 7 OS=Canis lupus familiaris OX=9615 GN=SLC12A7 PE=4 SV=2
F1PTV0	32	79364	1 (1)	1 (1)	Solute carrier family 15 member 1 OS=Canis lupus familiaris OX=9615 GN=SLC15A1 PE=3 SV=2
E2R578	25	34225	3 (1)	2 (1)	Solute carrier family 25 member 11 OS=Canis lupus familiaris OX=9615 GN=SLC25A11 PE=3 SV=2
J9NVP2	73	17292	2 (2)	1 (1)	Stathmin OS=Canis lupus familiaris OX=9615 GN=STMN1 PE=3 SV=1
E2RAU5	181	73904	3 (3)	2 (2)	Stress-70 protein, mitochondrial OS=Canis lupus familiaris OX=9615 GN=HSPA9 PE=3 SV=1
Q0QF09	140	61520	2 (2)	1 (1)	Succinate dehydrogenase (quinone) (Fragment) OS=Canis lupus familiaris OX=9615 GN=SDHA PE=2 SV=1
F1PJ13	186	57283	5 (3)	2 (2)	Succinyl-CoA:3-ketoacid-coenzyme A transferase OS=Canis lupus familiaris OX=9615 GN=OXCT1 PE=3 SV=2

Q8WNN6, F1Q462	225	16074	8 (8)	4 (4)	Superoxide dismutase [Cu-Zn] OS=Canis lupus familiaris OX=9615 GN=SOD1 PE=2 SV=1
E2RSF2	215	25001	6 (5)	4 (3)	Superoxide dismutase OS=Canis lupus familiaris OX=9615 GN=SOD2 PE=3 SV=1
F6XIK8	55	62845	3 (2)	2 (1)	Synaptotagmin binding cytoplasmic RNA interacting protein OS=Canis lupus familiaris OX=9615 GN=SYNCRIP PE=4 SV=1
E2R151	21	168867	3 (1)	2 (1)	Synemin OS=Canis lupus familiaris OX=9615 GN=SYNM PE=3 SV=2
F1PHP1	188	36849	4 (4)	3 (3)	Thioredoxin domain containing 5 OS=Canis lupus familiaris OX=9615 GN=TXNDC5 PE=3 SV=2
J9NWJ5	224	11665	6 (6)	4 (4)	Thioredoxin OS=Canis lupus familiaris OX=9615 PE=3 SV=1
F1PBX0	141	72481	5 (3)	4 (2)	Thioredoxin reductase 1 OS=Canis lupus familiaris OX=9615 GN=TXNRD1 PE=3 SV=2
J9P7X2	147	18257	5 (4)	3 (3)	Thy-1 cell surface antigen OS=Canis lupus familiaris OX=9615 GN=THY1 PE=4 SV=1
F1PAF0	30	54235	1 (1)	1 (1)	Tissue alpha-L-fucosidase OS=Canis lupus familiaris OX=9615 GN=FUCA1 PE=4 SV=2
A0A0A0MPC5	26	21595	38 (3)	1 (1)	Trafficking protein particle complex subunit 2 OS=Canis lupus familiaris OX=9615 GN=TRAPPC2 PE=4 SV=1
H9GW87	139	37678	3 (3)	2 (2)	Transaldolase OS=Canis lupus familiaris OX=9615 GN=LOC475937 PE=3 SV=1
E2RAE8	469	23656	11 (10)	5 (5)	Transgelin OS=Canis lupus familiaris OX=9615 GN=TAGLN PE=3 SV=2
F1P6P2	712	22534	13 (10)	6 (5)	Transgelin OS=Canis lupus familiaris OX=9615 GN=TAGLN2 PE=3 SV=2
F1PE28	753	63684	15 (14)	9 (8)	Transketolase OS=Canis lupus familiaris OX=9615 GN=TKT PE=4 SV=2
A0A0A0MPD0	1916	32085	40 (38)	16 (16)	Triosephosphate isomerase OS=Canis lupus familiaris OX=9615 GN=TPI1 PE=3 SV=1
F1P912, A0A0N9JIB9	100	37436	4 (2)	4 (2)	Tropomyosin 1 OS=Canis lupus familiaris OX=9615 GN=TPM1 PE=3 SV=1
F6X7L0, A0A0N9JE84	265	32903	11 (10)	7 (7)	Tropomyosin 4 OS=Canis lupus familiaris OX=9615 GN=TPM4 PE=3 SV=1
A0A077LQA5	125	50788	3 (3)	2 (2)	Tubulin alpha chain OS=Canis lupus familiaris OX=9615 GN=TUBA1A PE=2 SV=1
E2RNQ2	1568	57598	20 (20)	9 (9)	Tubulin alpha chain OS=Canis lupus familiaris OX=9615 GN=TUBA1C PE=3 SV=2
E2RBC3	120	50634	5 (2)	2 (1)	Tubulin alpha chain OS=Canis lupus familiaris OX=9615 GN=TUBA4A PE=3 SV=1
E2QSF4	1650	50095	29 (28)	12 (12)	Tubulin beta chain OS=Canis lupus familiaris OX=9615 GN=TUBB PE=3 SV=1
E2RFJ7, F1PQ68	1550	50377	25 (25)	11 (11)	Tubulin beta chain OS=Canis lupus familiaris OX=9615 GN=TUBB2A PE=3 SV=1
F2Z4P1	279	50856	6 (6)	3 (3)	Tubulin beta chain OS=Canis lupus familiaris OX=9615 GN=TUBB3 PE=3 SV=1

E2RFV2	146	50010	5 (4)	4 (3)	Tubulin beta chain OS=Canis lupus familiaris OX=9615 GN=TUBB4A PE=3 SV=2
L7N0I7	1496	50255	28 (25)	12 (11)	Tubulin beta chain OS=Canis lupus familiaris OX=9615 GN=TUBB4B PE=3 SV=1
E2QYC2	694	50291	19 (14)	10 (7)	Tubulin beta chain OS=Canis lupus familiaris OX=9615 GN=TUBB6 PE=3 SV=1
F1PBL1	211	27899	7 (5)	5 (3)	Tyrosine 3-monooxygenase/tryptophan 5-monooxygenase activation protein zeta OS=Canis lupus familiaris OX=9615 GN=YWHAZ PE=3 SV=2
E2RHR7	30	63383	1 (1)	1 (1)	Tyrosine--tRNA ligase OS=Canis lupus familiaris OX=9615 GN=YARS PE=3 SV=2
E2R311	41	55724	2 (2)	1 (1)	UDP-glucose 6-dehydrogenase OS=Canis lupus familiaris OX=9615 GN=UGDH PE=3 SV=1
F1PBJ8	304	57183	5 (5)	3 (3)	Uncharacterized protein OS=Canis lupus familiaris OX=9615 GN=ALDH2 PE=3 SV=2
J9P7A6	171	28692	5 (4)	3 (3)	Uncharacterized protein OS=Canis lupus familiaris OX=9615 GN=ALDOA PE=4 SV=1
E2R8S4	129	17546	2 (2)	1 (1)	Uncharacterized protein OS=Canis lupus familiaris OX=9615 GN=CALM2 PE=4 SV=2
F1Q0J0	587	23742	12 (12)	6 (6)	Uncharacterized protein OS=Canis lupus familiaris OX=9615 GN=GSTP1 PE=4 SV=2
E2R0T6	806	71082	23 (19)	14 (11)	Uncharacterized protein OS=Canis lupus familiaris OX=9615 GN=HSPA8 PE=3 SV=1
F1PTS8	144	60975	3 (3)	1 (1)	Uncharacterized protein OS=Canis lupus familiaris OX=9615 GN=KRT6A PE=3 SV=1
F1PVL5	160	57812	3 (3)	1 (1)	Uncharacterized protein OS=Canis lupus familiaris OX=9615 GN=KRT79 PE=3 SV=2
F1PCE8	108	26945	3 (3)	1 (1)	Uncharacterized protein OS=Canis lupus familiaris OX=9615 GN=LOC475521 PE=3 SV=1
F1Q0I8	169	23923	3 (2)	2 (1)	Uncharacterized protein OS=Canis lupus familiaris OX=9615 GN=LOC476006 PE=4 SV=2
F6V1W9	130	112612	5 (4)	2 (2)	Uncharacterized protein OS=Canis lupus familiaris OX=9615 GN=LOC477072 PE=4 SV=1
E2REA4	94	27498	4 (3)	2 (2)	Uncharacterized protein OS=Canis lupus familiaris OX=9615 GN=LOC479459 PE=3 SV=2
F1P6R7	97	25863	2 (2)	1 (1)	Uncharacterized protein OS=Canis lupus familiaris OX=9615 GN=LOC479912 PE=4 SV=2
F6USN4	49	165213	6 (4)	2 (1)	Uncharacterized protein OS=Canis lupus familiaris OX=9615 GN=LOC611455 PE=4 SV=1
E2R5M5	142	23370	2 (2)	1 (1)	Uncharacterized protein OS=Canis lupus familiaris OX=9615 GN=NACA PE=4 SV=1
E2R268	197	39566	2 (2)	1 (1)	Uncharacterized protein OS=Canis lupus familiaris OX=9615 GN=PDHB PE=4 SV=1
E2RHG2	287	22338	12 (10)	6 (5)	Uncharacterized protein OS=Canis lupus familiaris OX=9615 GN=PRDX1 PE=4 SV=1
E2RRD4	63	28372	5 (5)	2 (2)	Uncharacterized protein OS=Canis lupus familiaris OX=9615 GN=PRDX3 PE=4 SV=1
E2R9Y9	100	11699	2 (2)	1 (1)	Uncharacterized protein OS=Canis lupus familiaris OX=9615 GN=RPLP2 PE=3 SV=1

F1PEZ4	365	18296	8 (8)	3 (3)	Uncharacterized protein OS=Canis lupus familiaris OX=9615 GN=RPS27A PE=4 SV=2
E2R995	34	24661	2 (2)	1 (1)	Uncharacterized protein OS=Canis lupus familiaris OX=9615 GN=WDR31 PE=4 SV=2
J9P314	42	47284	1 (1)	1 (1)	Uncharacterized protein OS=Canis lupus familiaris OX=9615 PE=3 SV=1
F1P8Z1	29	22080	1 (1)	1 (1)	Uncharacterized protein OS=Canis lupus familiaris OX=9615 PE=3 SV=2
J9NSG1	65	10985	2 (2)	1 (1)	Uncharacterized protein OS=Canis lupus familiaris OX=9615 PE=4 SV=1
J9NY83	20	50688	2 (2)	1 (1)	Uncharacterized protein OS=Canis lupus familiaris OX=9615 PE=4 SV=1
J9P558	68	14594	2 (2)	1 (1)	Uncharacterized protein OS=Canis lupus familiaris OX=9615 PE=4 SV=1
E2R4V7	129	28359	2 (2)	1 (1)	Uncharacterized protein OS=Canis lupus familiaris OX=9615 PE=4 SV=2
E2R8Y8	23	52022	3 (1)	1 (1)	Uncharacterized protein OS=Canis lupus familiaris OX=9615 PE=4 SV=2
E2REX9	642	32534	6 (6)	2 (2)	Uncharacterized protein OS=Canis lupus familiaris OX=9615 PE=4 SV=2
F1P7X4	51	11667	3 (2)	1 (1)	Uncharacterized protein OS=Canis lupus familiaris OX=9615 PE=4 SV=2
F1PNI3	150	39949	4 (4)	2 (2)	Uncharacterized protein OS=Canis lupus familiaris OX=9615 PE=4 SV=2
H9GWE2	606	34030	12 (12)	5 (5)	Uncharacterized protein OS=Canis lupus familiaris OX=9615 PE=4 SV=2
A0A0E3ZNN3	28	35769	2 (1)	1 (1)	Uncharacterized protein OS=Pasteurella multocida subsp. multocida OH4807 OX=1304873 GN=I926_03315 PE=4 SV=1
E2RLQ9	127	91640	5 (5)	2 (2)	Valosin containing protein OS=Canis lupus familiaris OX=9615 GN=VCP PE=3 SV=2
F1PLS4	421	53622	10 (9)	6 (6)	Vimentin OS=Canis lupus familiaris OX=9615 GN=VIM PE=3 SV=1
F1PR93	43	67007	2 (2)	1 (1)	WD repeat domain 1 OS=Canis lupus familiaris OX=9615 GN=WDR1 PE=2 SV=2

Supplementary Table S7. Primary antibodies used in immunofluorescence.

Antibody	Manufacture	Especificity	Clone	Diluition	Secundary antibody
PTEN	ABGENT, San Diego, CA, USA	NA	Bs-0686R	1: 500	Rabbit
p-AKT	Cell Signaling, Danvers, MA, USA	Ser473	D9E	1: 100	Rabbit
p-mTOR	Cell Signaling, Danvers, MA, USA	Ser2448	49F9	1: 100	Rabbit
p-4EBP1	Cell Signaling, Danvers, MA, USA	Thr37/46	NA	1: 100	Rabbit

* NA = not applicable

Supplementary Table S8. Sequence of oligonucleotides from genes used for RT-qPCR.

Access gene symbol*	Oligunucleotides sequence (5'>3')
HPRT	*Forward primer (5'3')* AGCTTGCTGGTGAAAAGGAC
	Reverse primer (3'5') TTATAGTCAAGGGCATATCC
RPS19	*Forward primer (5'3')* CCTTCCTCAAAAAGTCTGGG
	Reverse primer (3'5') GAACGAGGGATGCTACTCTTG
RPS5	*Forward primer (5'3')* TCACTGGTGAGAACCCCCT
	Reverse primer (3'5') TCACTGGTGAGAACCCCCT
PTEN	*Forward primer (5'3')* CGACGGGAAGACAAGTTCATG
	Reverse primer (3'5') TCACCGCACACAGGCAAT
mTOR	*Forward primer (5'3')* CTGGCCGGATGTAAACGAA
	Reverse primer (3'5') GCGTATCGATTCTCGCAATGA
AKT	*Forward primer (5'3')* TTCTACGGCGCCGAGATC
	Reverse primer (3'5') TTCTTGCACCAGATGGCGCT

*GenBank (www.ncbi.nlm.nih.gov)
